# The Polyfunctionality of Human Memory CD8+ T Cells Elicited by Acute and Chronic Virus Infections Is Not Influenced by Age

**DOI:** 10.1371/journal.ppat.1003076

**Published:** 2012-12-13

**Authors:** Alina Lelic, Chris P. Verschoor, Mario Ventresca, Robin Parsons, Carole Evelegh, Dawn Bowdish, Michael R. Betts, Mark B. Loeb, Jonathan L. Bramson

**Affiliations:** 1 McMaster Immunology Research Centre, Department of Pathology and Molecular Medicine, McMaster University, Hamilton, Ontario, Canada; 2 Department of Mechanical and Industrial Engineering, University of Toronto, Toronto, Ontario, Canada; 3 Department of Microbiology, University of Pennsylvania, Philadelphia, Pennsylvania, United States; Oregon National Primate Research Center, United States of America

## Abstract

As humans age, they experience a progressive loss of thymic function and a corresponding shift in the makeup of the circulating CD8+ T cell population from naïve to memory phenotype. These alterations are believed to result in impaired CD8+ T cell responses in older individuals; however, evidence that these global changes impact virus-specific CD8+ T cell immunity in the elderly is lacking. To gain further insight into the functionality of virus-specific CD8+ T cells in older individuals, we interrogated a cohort of individuals who were acutely infected with West Nile virus (WNV) and chronically infected with Epstein Barr virus (EBV) and Cytomegalovirus (CMV). The cohort was stratified into young (<40 yrs), middle-aged (41–59 yrs) and aged (>60 yrs) groups. In the aged cohort, the CD8+ T cell compartment displayed a marked reduction in the frequency of naïve CD8+ T cells and increased frequencies of CD8+ T cells that expressed CD57 and lacked CD28, as previously described. However, we did not observe an influence of age on either the frequency of virus-specific CD8+ T cells within the circulating pool nor their functionality (based on the production of IFNγ, TNFα, IL2, Granzyme B, Perforin and mobilization of CD107a). We did note that CD8+ T cells specific for WNV, CMV or EBV displayed distinct functional profiles, but these differences were unrelated to age. Collectively, these data fail to support the hypothesis that immunosenescence leads to defective CD8+ T cell immunity and suggest that it should be possible to develop CD8+ T cell vaccines to protect aged individuals from infections with novel emerging viruses.

## Author Summary

The prevalence and severity of viral infections increases with advanced age, a phenomenon associated with a defective immune system. The thymic output of naïve T cells declines as we age and it is this lack of naïve T cells that is believed to contribute to the inability of the aged to respond to novel infections and develop subsequent memory T cell responses. Here we show that individuals aged 60+ are capable of developing memory CD8+ T cells to West Nile virus, novel pathogen, indistinguishable in terms of polyfunctionality to those of subjects <60 years of age. Furthermore, we show that chronic and life-long infections with CMV and EBV result in similar polyfunctional virus-specific memory CD8+ T cell responses in subjects of all age groups. Our work demonstrates that aged individuals can elicit functional memory CD8+ T cell responses to a new pathogen while maintaining polyfunctional CD8+ T cells against recurrent chronic virus infections. Current vaccine platforms, which rely upon inactivated pathogens or recombinant subunits, are poorly effective in the aged. Our data suggest that live viruses may be more effective vaccine platforms in older humans.

## Introduction

CD8+ T cells can provide robust protection against pathogens and tumors. As a result, significant effort has been invested into developing vaccines that elicit protective CD8+ T cell memory responses. It is generally believed that immunological function decreases with advanced age, a phenomenon known as *immunosenescence*, rendering older individuals at higher risk of infection. While vaccination would seem to be an appropriate intervention to improve protective immunity, several reports have demonstrated that older individuals mount impaired responses to conventional vaccines, suggesting that alternate platforms or strategies may be required. Notably, antibody responses to influenza and tick-borne encephalitis vaccines were impaired in the elderly [1], [2], [3]. With regard to T cell immunity, recent reports from a large-scale immunization study with a live vaccine against varicella zoster demonstrated that while it is possible to boost zoster-specific CD4+ T cells to a protective level in individuals >60 years of age, vaccine responsiveness did appear to wane in individuals >75 years of age [4], [5]. These data support the concept that immunosenescence may be an issue to overcome in the development of effective vaccines for elderly individuals; however, further research is required to truly understand the extent of immune dysfunction in older humans.

Alterations in the CD8+ T cell compartment are among the most common characteristics in the elderly T cell repertoire and are thought to reflect an impaired ability to control infection [6], [7]. The aged CD8+ T cell population is characterized by a high proportion of CD28− cells (often co-expressing NK markers, such as CD57), which are believed to reflect highly differentiated T cells that lack the capacity to proliferate [1], [8]. In some cases, the CD8+ CD28− T cell population comprises an oligoclonal expansion of CMV-reactive cells, suggesting that chronic infections may preoccupy the immune response in the elderly, leading to a CD8+ T cell repertoire with limited diversity [9], [10], [11], [12]. The sum of these observations suggests that the CD8+ T cell population in the elderly is compromised in its capacity to respond to novel infections. However, the exact relationship between the global phenotypic changes in the CD8+ T cell compartment that appear with age and the functionality of antigen-specific CD8+ T cells is poorly defined. Further, there is a paucity of data regarding the ability of the elderly to mount CD8+ T cell responses to novel infections. Although it is generally assumed that age-associated changes in the CD8+ T cell compartment may explain the heightened risk of elderly individuals to infection, experimental data are sparse. Herein we provide one of the few studies in humans that demonstrate the impact of age on CD8+ T cell immunity to pre-existing and novel viral infections.

West Nile virus (WNV) emerged as a novel human pathogen in the Northern hemisphere in 1999, and since then has caused numerous viral outbreaks across North America [13], [14], [15]. From 2003–2008, we collected sequential blood specimens from >100 people acutely infected with WNV with an age distribution ranging from 19–85 years. Given this age range, we reasoned that our cohort would be suitable to study the relationship between age and the development of virus-specific CD8+ T cells following a novel acute infection. In our original report of this cohort, we observed that age did not influence the magnitude or breadth of the memory T cell response to WNV [16], suggesting that age may not impair the development of CD8+ T cell immunity against acute infections.

Our previous work did not address the longevity or functionality of CD8+ T cell memory that develops following WNV infection. Thus, it remained possible that the older members of our cohort failed to develop a CD8+ T cell memory pool that was functionally equivalent to the younger members. In this current report, we have examined the polyfunctionality of the WNV-reactive CD8+ T cell population at later time points post-infection. We have also examined memory responses to EBV and CMV within this cohort, as these lifelong infections may differentially impact the functionality of memory CD8+ T cells. Our results reveal that although the memory CD8+ T cells display distinct polyfunctional states that are virus-specific, we observed no impact of ageing on polyfunctionality. These studies have revealed that memory CD8+ T cell immunity in older individuals is intact and suggest that vaccine development should focus on other parameters that may be defective in the elderly.

## Results

### Ageing results in increased frequencies of highly-differentiated CD8+ T cells and decreased frequencies of naïve CD8+ T cells

**Figure 1:**
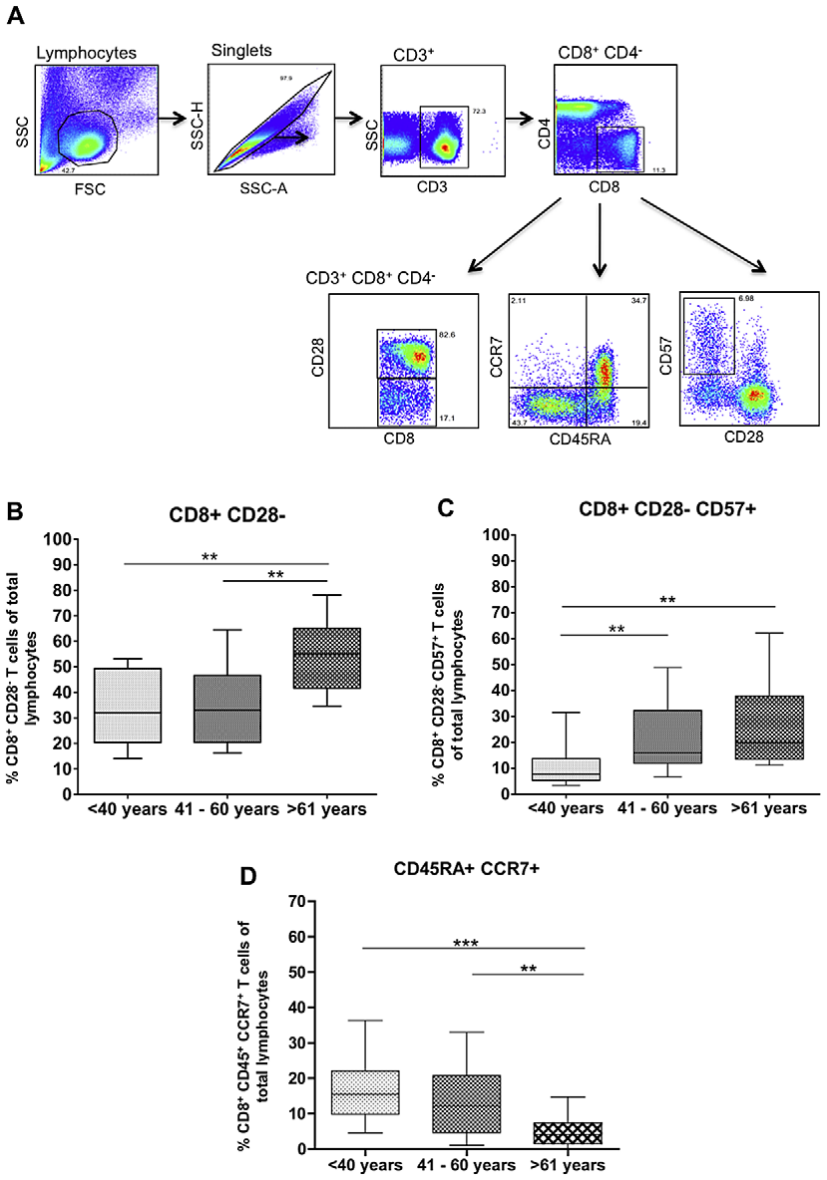
Ageing results in increased frequencies of highly-differentiated CD8+ T cells and decreased frequencies of naïve CD8+ T cells. A) Flow plots depicting gating strategy for phenotypic analysis. B) Percentage of CD8+ CD28− T cells within the peripheral blood lymphocyte pool; C) Percentage of CD8+ CD28− CD57+ T cells (terminally differentiated cells) within the peripheral blood lymphocyte pool; D) Percentage of CD45RA+ CCR7+ (naïve) T cells within the peripheral blood lymphocyte pool. Data are group according to stratification described in the Materials and Methods. Statistical analysis perfomed by one-way ANOVA with Tukey's mulitple comparison post-test. Box and whiskers plots are calculated at 95% confidenced interval.

For these studies, we have examined the CD8+ T cell memory responses from a cohort of 72 patients who were naturally infected with West Nile virus (WNV). We stratified our cohort into 3 groups: *young* (<40 years of age; n = 21), *middle-aged* (41–59 years of age; n = 25) and *aged* (>60 years of age; n = 26). To confirm that these cohorts displayed the expected age-associated changes in the CD8+ T cell compartment, we compared the phenotype of CD8+ T cells among the three different age groups. Significantly higher frequencies of CD8+ CD28− and CD8+ CD28− CD57+ cells were observed within the aged cohort (Figure 1A and 1B). Likewise, we noted that the CD45RA+ CD28+ CD8+ T cell population was significantly decreased in the naïve T cell pool in middle-aged and aged populations compared to the young population (Figure 1C). We also observed a significant reduction in the presence of naïve (CD45RA+ CCR7+) T cells in the aged subjects (Figure 1D). These observations confirm that our aged cohort displayed the expected immunosenescent phenotype within the CD8+ T cell compartment.

### Age does not impact the frequency of functional virus-specific CD8+ T cells

**Figure 2:**
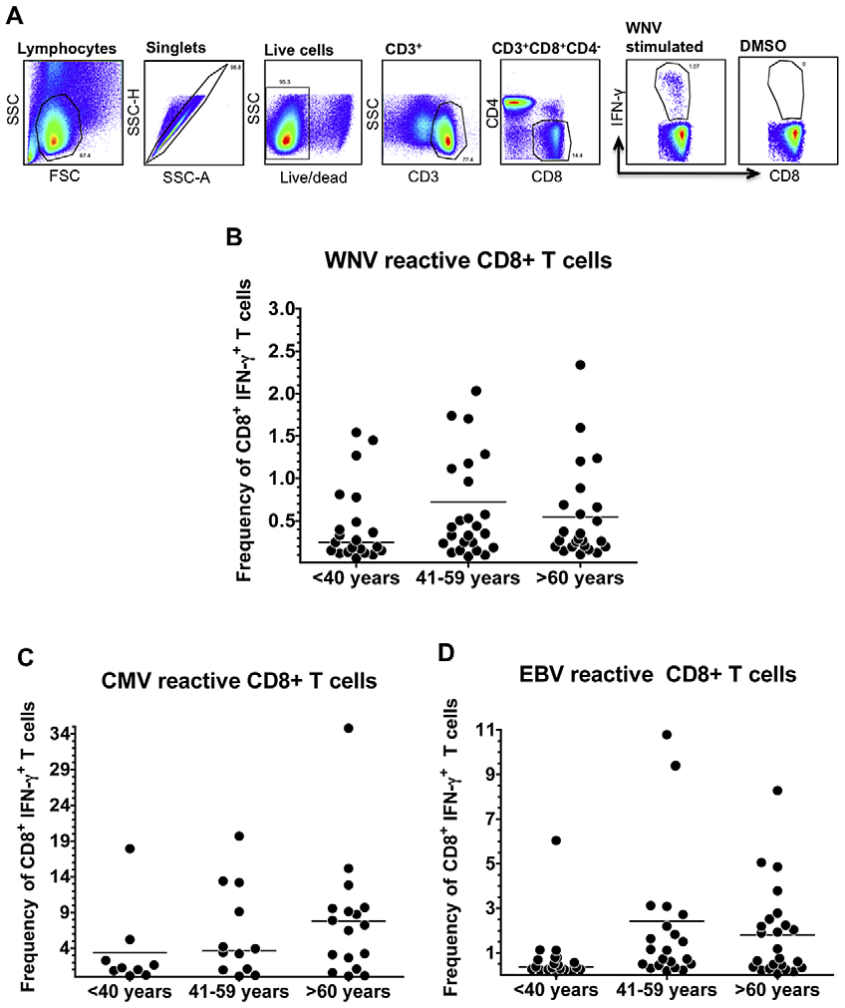
Age does not impact the frequency of functional virus-specific CD8+ T cells. A) Flow plots depict gating strategy for IFNγ+ CD8+ T cells. (B–D) Pooled WNV, CMV or EBV peptides were used to stimulated freshly thawed PBMCs isolated from WNV-naturally infected subjects 6–7 months post symptom onset. Scatter plots depict IFNγ+ CD8+ T cell responses only from reactive subjects (>0.05% and 3 fold above DMSO background); (B) WNV reactive subjects: 20/21 Young, 24/25 Mid-aged, 24/26 Aged; (C) CMV reactive subjects: 7/21 Young, 12/25 Mid-aged, 17/26 Aged; (D) EBV reactive subjects: 17/21 Young, 21/25 Mid-aged, 24/26 Aged. Means are displayed as horizontal lines. Statistical analysis performed by one-way ANOVA followed by Tukey's multiple comparison post-test.

We first sought to confirm our previous results showing that age did not impact the magnitude of the WNV-specific CD8+ T cell response. In our original study, we employed ELISPOT to monitor WNV-specific CD8+ T cells. However, cytokine production by ELISPOT cannot be attributed solely to CD8+ T cells. Therefore, in the current study, we employed flow cytometry to specifically identify cytokine-producing CD8+ T cells and provide a more accurate assessment of the functionality of the virus-specific CD8+ T cells. For these experiments, we used specimens obtained 6–7 months following WNV infection. Since our study population consisted of individuals with diverse HLAs, virus-specific CD8+ T cells were identified based on cytokine production following stimulation with a broad collection of immunodominant peptides that span the breadth of HLAs expressed by our cohort. Briefly, for these experiments, freshly thawed PBMCs were stimulated with pools of dominant epitope peptides derived from WNV, CMV or EBV and cytokine production (IFN-γ, TNF-α and IL-2) was measured on a per-cell basis using flow cytometry. While the CD8+ T cells produced varying amounts of cytokine following peptide stimulation, we did not observe any peptide-specific CD8+ T cells that could produce IL-2 or TNF-α in the absence of IFN-γ. Since all of our peptide-stimulated CD8+ T cells expressed IFN-γ, which is considered to be the cytokine that mediates the primary anti-viral response by the adaptive immune system [17], we defined “virus-specific” CD8+ T cells as those which produced IFN-γ following stimulation with specific peptide epitopes (see Table S1 for a complete list of measured IFN-γ frequencies). The frequencies of CD8+ T cells specific for either WNV, CMV or EBV was similar among all age groups (Figure 2). We did note a trend towards elevated frequencies of CMV- and EBV-specific CD8+ IFN-γ+ T cells in the middle-aged and aged cohorts relative to the young cohort, but this trend did not reach statistical significance (Figures 2B–D).

### Stability of CD8+ T cell memory following acute infection is not influenced by age

While our data indicate that older individuals mount CD8+ T cell responses to acute infection (i.e. WNV) that are equivalent in magnitude to younger individuals, it is possible that the responses display different stability. To address this question, we measured the frequencies of WNV-specific CD8+ T cells in our cohort at 2 additional time points: baseline (early memory; average of 35 days after symptom onset) and 2–4 years post symptom onset (late memory). The magnitude of the WNV-specific CD8+ T cell response was highest at 1 month and declined thereafter (Table 1). Importantly, the magnitude of the WNV-specific CD8+ T cells was equivalent among the various age cohorts at all 3 time points, suggesting that the longevity of the memory CD8+ T cell response is not age-dependent.

**Table 1:** Stability of WNV-specific CD8+ T cell memory response is not influenced by age^1^.

	Young (<40 years of age)	Middle-aged (41–59 years)	Aged (>60 years of age)
***BASELINE***			
**% WNV-specific CD8^+^ T cells** 2	0.4102±0.3057	0.5553±0.6462	0.6118±0.7584
**% TNFα^+^ of total CD8^+^ IFNγ^+^ T cells** 3	35.69±11.87	42.11±17.82	39.22±16.85
**% IL2^+^ of total CD8^+^ IFNγ^+^ T cells** 4	3.363±3.564	1.565±1.416	2.635±2.213
***6–7 MONTHS***			
**% WNV-specific CD8^+^ T cells** 2	0.3577±0.3743	0.4158±0.5169	0.3260±0.3082
**% TNFα^+^ of total CD8^+^ IFNγ^+^ T cells** 3	47.51±17.17	56.59±19.85	42.95±15.61
**% IL2^+^ of total CD8^+^ IFNγ^+^ T cells** 4	1.595±1.589	2.713±1.613	1.977±1.872
***>2 YEARS***			
**% WNV-specific CD8^+^ T cells** 2	0.157±0.21	0.094±0.06	0.119±0.09
**% TNFα^+^ of total CD8^+^ IFNγ^+^ T cells** 3	34.94±14.50	41.22±19.39	29.90±15.38
**% IL2^+^ of total CD8^+^ IFNγ^+^ T cells** 4	3.698±2.125	2.639±3.274	2.822±2.307

1 All data represent the mean +/− standard deviation.

2 Percentage of CD3+CD8+ cells that produce cytokine in response to WNV peptides.

3 Percentage of CD3+CD8+ IFNγ+ cells that also produce TNFα.

4 Percentage of CD3+CD8+ IFNγ+ cells that also produce IL2.

For these experiments, we also examined the production of TNF-α and IL-2 following peptide stimulation. As stated above, we did not observe any CD8+ T cells that produced TNF-α or IL-2 in the absence of IFN-γ following WNV peptide stimulation. We observed that 30%–50% of the WNV-specific CD8+ T cells were IFN-γ+ TNF-α+ double positive (Table 1). We also noted that only a fraction of WNV-specific CD8+ T cells could produce IL-2 and this did not increase with time. No difference was observed in the frequencies of TNF-α- or IL-2-producing WNV-specific CD8+ T cells among the 3 age groups at any time point (Table 1).

### Defining polyfunctional T cell populations using FLOCK

**Figure 3:**
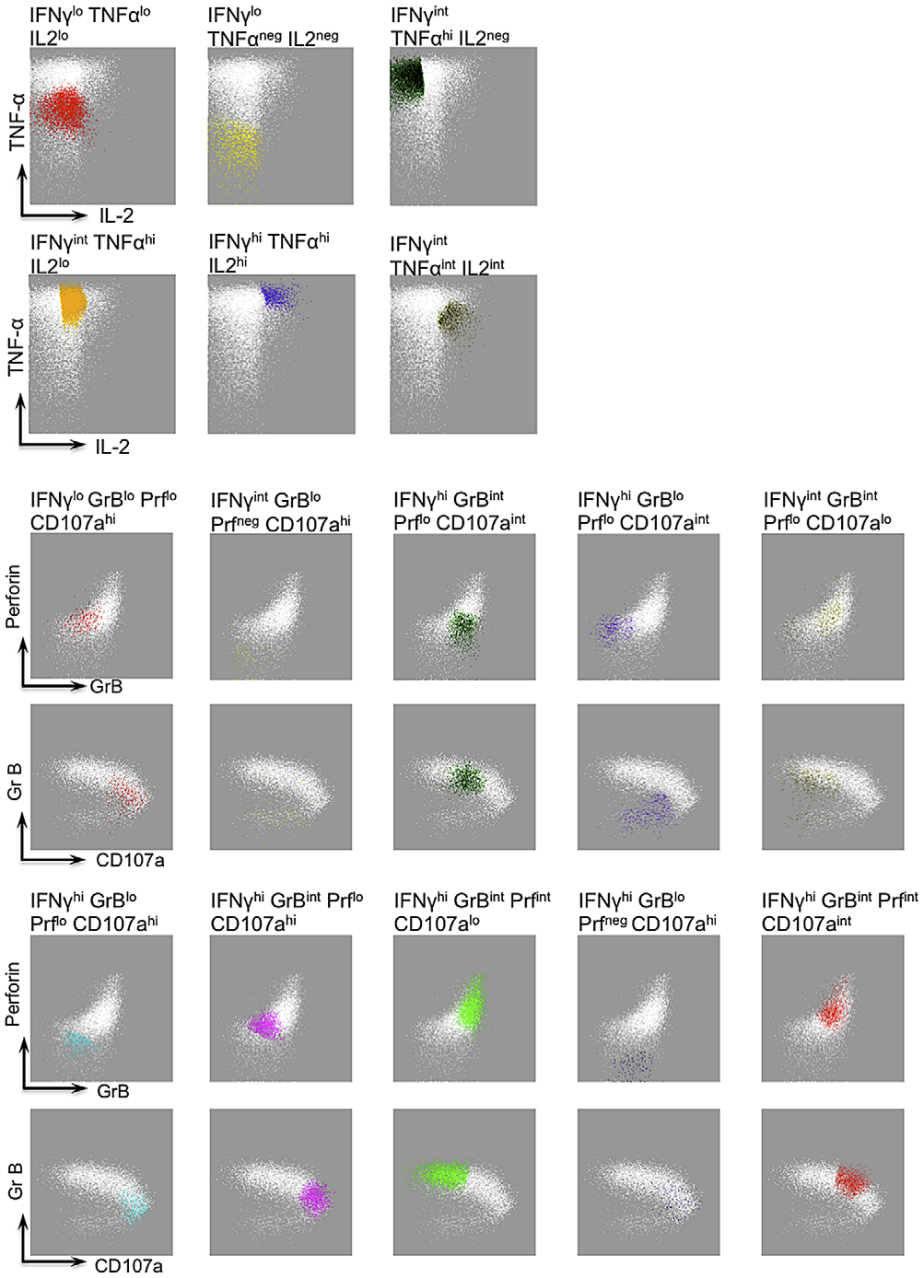
FLOCK gating strategy and description of IFN-g functional populations. A single representative sample, stimulated with CMV peptide pool, depicts the gating strategy by FLOCK, which identified the 16 functional populations (shown in various different colors and specified by title above the plot). WHITE dots in each panel depict the IFNγ+ CD8+ T cells and the functional populations (depicted by colours) are defined based on the level of expression of IFNγ and other cytokines (TNFα and IL2) or cytotoxic molecules (CD107a, GrB, and Perforin) as shown by the arrows. The same functional populations were applied in cross-sample comparison of WNV, CMV and EBV-specific CD8+ T cells.

In the previous paragraph, WNV-specific CD8+ T cells were crudely separated into 3 populations based on the expression of either IFN-γ, TNF-α, or IL-2. To gain further insight into the polyfunctional nature of the virus-specific CD8+ T cells, we also measured the cytotoxic capacity of the CD8+ T cells by granzyme B expression, upregulation of perforin and mobilization of CD107a (a measure of degranulation) following peptide stimulation of WNV samples obtained 6–7 months post symptom onset. Similar to our observations with cytokine production, we did not observe any peptide-specific CD8+ T cells that could upregulate perforin or mobilize CD107a in the absence of IFN-γ. Therefore, all functional parameters have been defined relative to the expression of IFN-γ. Polyfunctionality of antigen-specific CD8+ T cells was defined using a newly developed computational analysis of flow cytometry data: FLOCK (FLOw Cytometry without K), publicly available in the Immunology Database and Analysis Portal – ImmPort (www.immport.org). FLOCK utilizes a density-based clustering approach and algorithms to define biologically relevant populations from multiparametric data sets without the bias of manual gating [18]. Using FLOCK, we identified 16 distinct functional populations from IFN-γ+ CD8+ T cells for all three antigens (WNV, CMV and EBV), that were defined as negative (neg), low (lo), intermediate (int), and high (hi), based on the signal intensity of each marker (Figure 3). All populations were derived from IFN-γ+ events, thus there were no IFN-γ negative events.

**Figure 4:**
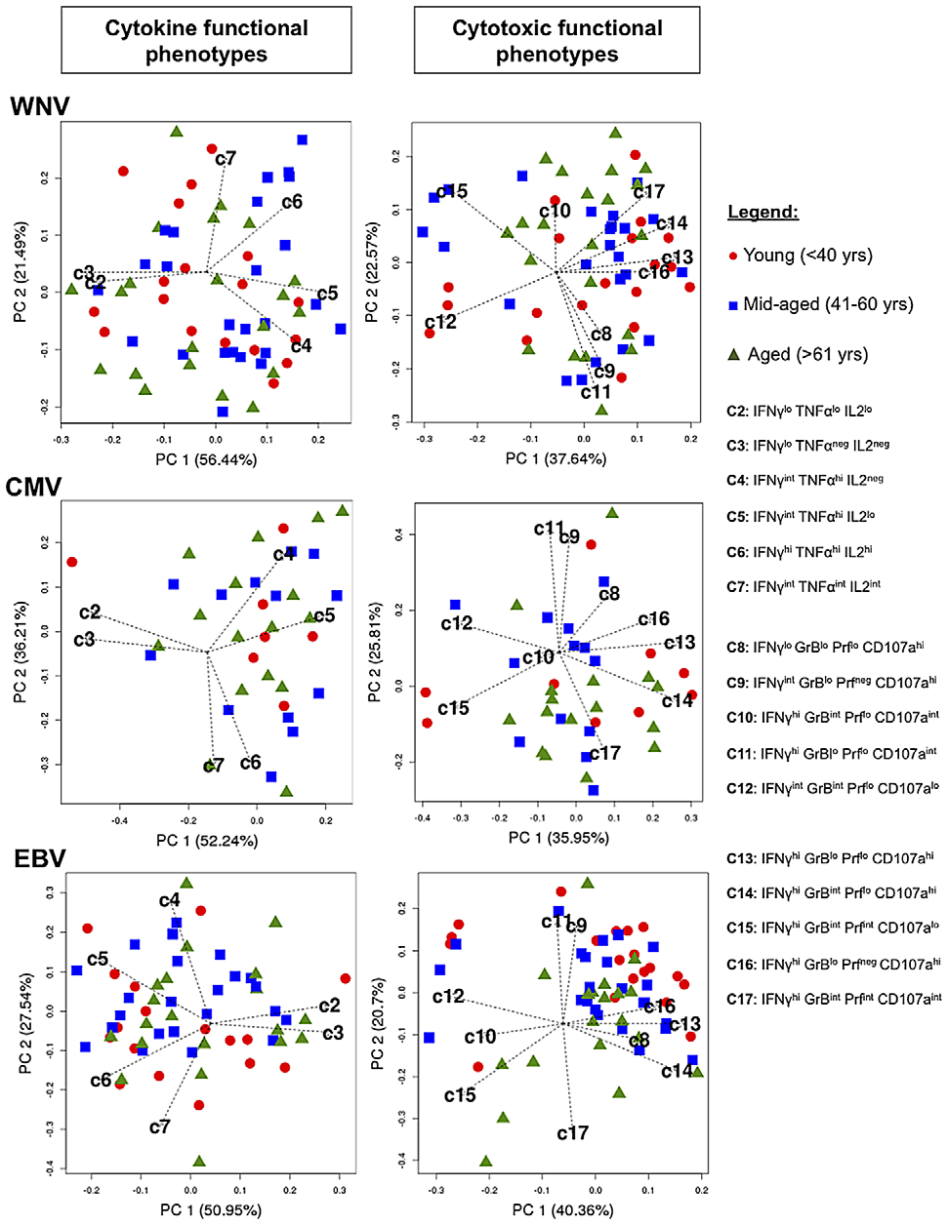
Principal Component Analysis of antigen-specific CD8+ T cell polyfunctionality shows no correlation with age. The Principal Component Analysis plots display biplots of top −2 principal components. The biplots show the samples (red circles = Young <40 years, blue squares = Mid-aged 41–59 years, green triangles = Aged >60 years) and the FLOCK defined functional CD8+ T cell phenotypes as a vector in a two-dimensional plane. The length of each vector indicates the approximate variance of the specific functional population. Lack of clustering of data points around specific vectors signifies lack of correlation between age and functionality of CD8+ T cells. Principal Component Analysis done by R version 2.14. ***PC1***, principal component 1; ***PC2***, principal component 2.

To address the question of whether advanced age impacted the development of polyfunctional memory CD8+ T cell responses, we analyzed the large data set comprising functional population frequencies (FLOCK identified) by Principal Component Analysis (PCA). PCA is a linear technique that transforms data of interrelated variables into a set of uncorrelated principal components (PCs) while maintaining the original variation of the data set in reduced dimensionality [19]. The polyfunctional analysis of antigen-specific CD8+ T cells was separated into two parameters: cytokine functional populations (C2–C7; Figure 4) and cytotoxic functional populations (C8–C17; Figure 4). Consequently, two PCA analyses were generated per antigen. PCA plots comprising cytokine functional populations for WNV, CMV and EBV were generated using the top two PCs that accounted for 78%, 86% and 78% of the overall variance, respectively. Functional populations C2 (IFNγ^lo^ TNFα^lo^ IL2^lo^) and C3 (IFNγ^lo^ TNFα^neg^ IL2^neg^); C4 (IFNγ^int^ TNFα^hi^ IL2^neg^) and C5 (IFNγ^int^ TNFα^hi^ IL2^lo^); C6 (IFNγ^hi^ TNFα^hi^ IL2^hi^) and C7 (IFNγ^int^ TNFα^int^ IL2^int^) tended to cluster, indicating a strong positive correlation (Figure 4). The vector clustering would suggest that these functional populations are the same or very similar. We observed no specific age clustering, suggesting no relationship between age and CD8+ T cell function based on cytokine production.

PCA plots depicting the cytotoxic functional phenotypes for WNV, CMV and EBV- specific CD8+ T cells were generated using the top two PCs and account for roughly 65% of the overall variance (Figure 4). It is important to note that the interpretation of the data did not change when we examined 3-D plots of the first 3 PCs (approximately 80% of the total variance) and for simplicity of interpretation we used biplots to explain these data. We observed high positive correlations between populations C9 (IFNγ^int^ GrB^lo^ Prf^neg^ CD107a^hi^) and C11 (IFNγ^hi^ GrB^lo^ Prf^lo^ CD107a^int^); C13 (IFNγ^hi^ GrB^lo^ Prf^lo^ CD107a^hi^) and C16 (IFNγ^hi^ GrB^lo^ Prf^neg^ CD107a^hi^), which suggests that they might belong to the same functional population but were segregated in into two based on automated binning by FLOCK analysis. We also find that population C10 (IFNγ^hi^ GrB^int^ Prf^lo^ CD107a^int^) contributes very little to the overall variance of the system for WNV and CMV since its vector length is small relative to the other defined cytotoxic phenotypes. Furthermore, as observed for antigen-specific CD8+ T cell cytokine function, we found no evidence that CD8+ T cell cytotoxicity was affected by age. These results suggest that while different combinations of cytotoxic markers define virus-specific CD8+ T cell responses, they show no linear relationship with age.

### CD8+ T cell polyfunctionality is virus-specific

**Figure 5:**
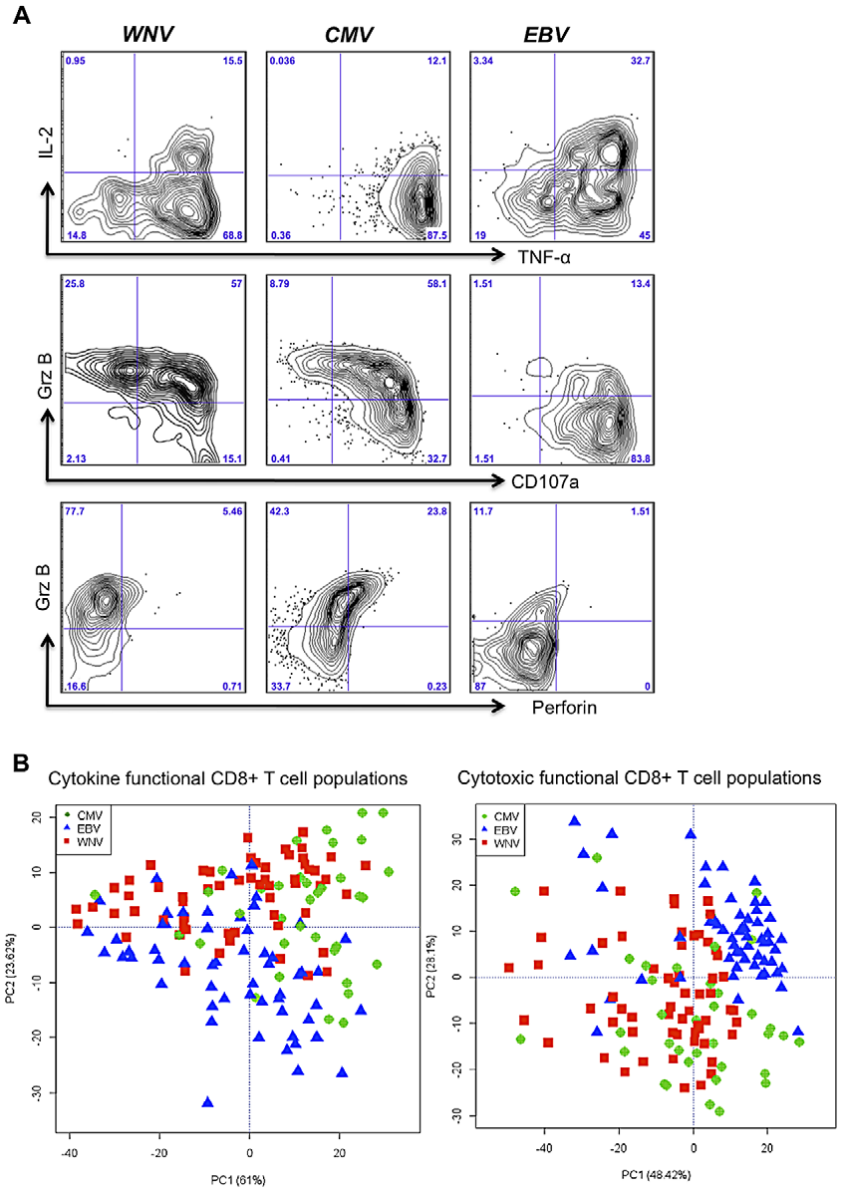
CD8+ T cell polyfunctionality is virus-specific. A) Flow plots depict representative samples following WNV, CMV, and EBV peptide stimulations and the cytokine and cytotoxic marker staining on IFNγ+ CD8+ T cells. B) Principal component analysis of FLOCK identified cytokine and cytotoxic functional phenotypes shows clustering of virus-specific functional T cells that are similar between WNV and CMV but different for EBV. Principal Component Analysis done by R version 2.14. ***PC1***, principal component 1; ***PC2***, principal component 2.

Preliminary analysis of polyfunctional WNV, CMV, and EBV-specific IFN-γ+ CD8+ T cells (producing cytokines; IL2 and TNF-α, and mobilizing cytotoxic mediators; GrB, perforin and CD107a) revealed that WNV and CMV polyfunctional responses were more similar than EBV-specific polyfunctional CD8+ T cells (Figure 5A). On average, EBV-specific CD8+ T cells were better producers of IL2 but failed to upregulate Granzyme B or perforin in comparison to WNV and CMV-specific CD8+ T cells (Figure 5A). We next performed the Kolmogorov-Smirnov (KS) test to determine whether WNV, CMV or EBV-specific CD8+ T cell functional phenotypes defined by FLOCk were drawn from the same distributions. The KS test is based on the null hypothesis that the samples are drawn from the same distribution, thus larger p-values suggest that the two sets are similar. A comparison of the evaluated KS statistics between the different viral antigens for all functional phenotypes showed that WNV and CMV were more functionally similar than EBV (Figure 5B). For example, T cell phenotype identified in population C4 (IFNγ^int^ TNFα^hi^ IL2^neg^) for WNV and CMV had a calculated p value of 0.889 suggesting a very similar distribution of this functional population, which was not observed for EBV. We further noted that more than 40% of the functional cells (IFNγ+) simultaneously produced TNFα+ for all three antigens, but there was a difference in the ability to produce IL-2. WNV-specific CD8+ T cells produced the least IL-2, EBV-specific CD8+ T cells produced the highest amounts of IL-2 and CMV-specific CD8+ T cells displayed an intermediate phenotype (Figure 5A).

The KS analysis of the cytotoxic functional populations revealed a striking similarity between CMV- and WNV-specific T cells, where populations C8 (IFNγ^lo^GrB^lo^Prf^lo^CD107a^hi^) and C16 (IFNγ^hi^GrB^lo^Prf^neg^CD107a^hi^) were distributed similarly (p = 0.909 and p = 0.882, respectively). Overall, a higher frequency of CMV- and WNV-specific memory T cells were cytotoxic and polyfunctional (GrB+ Prf+ CD107a+) in comparison to the EBV-specific T cells, which became CD107a^hi^ following peptide stimulation but remained low in terms of GrB and Perforin expression (Figure 5A).

Using PCA biplots, we were able to discriminate antigenic stimulation (WNV, CMV or EBV) based on the resultant functional phenotypes (Figure 5B). Corroborating the KS distribution analysis, the PCA showed that CMV and WNV are indiscriminant based on the above-mentioned functional populations, whereas EBV-specific functional phenotypes cluster separately. This effect of EBV segregating away from WNV and CMV was especially evident when cytotoxic populations were analyzed by PCA (Figure 5B). Altogether, it does not appear that age has an impact on the development of memory CD8+ T cells with the capacity to elaborate multiple functions. Rather, it appears that the polyfunctional profile of virus-specific CD8+ T cells appears to be a function of the pathogen.

## Discussion

Contrary to the suggestion that susceptibility to new infections in the aged occurs due to insufficient CD8+ T cell immunity as a result of diminished frequencies of naïve CD8+ T cells and/or dysfunctional CD8+ T cell memory [20], [21], we have shown that aged individuals mount CD8+ T cell memory responses to a novel viral agent that are equivalent to young individuals. In fact, extensive analysis of CD8+ T cell functional parameters revealed no relationship between age and the capacity to produce cytokines or mobilize cytotoxic mediators in response to stimulation by peptides derived from viruses responsible for both acute (WNV) and chronic (CMV, EBV) infections despite clear evidence of an immunosenescent phenotype in the bulk CD8+ T cell pool (elevated frequencies of CD28− CD57+ cells and decreased frequencies of CD45RA+ CCR7+ cells relative to the younger members of the cohort). Thus, although the members of our aged cohort displayed expected age-related changes in the composition of the CD8+ T cell compartment, these alterations did not manifest as a defect in functional virus-specific immunity, even when the primary virus infection occurred in old age, as in the case of WNV.

A recent study revealed that infection of middle-aged and old macaques with Rhesus CMV (RhCMV) produced RhCMV-specific CD8+ T cells with comparable functionality in both age groups [22], supporting the concept that anti-viral CD8+ T cell responses may not be dysfunctional in aged individuals. In contrast, immunization with modified vaccinia Ankara (MVA) elicited weaker CD8+ T cell responses in old macaques compared to young macaques [7]. While the results of the MVA experiments may seem at odds with our observations, the authors of this latter report employed live vaccinia virus to stimulate MVA-specific CD8+ T cells *in vitro* for their functional assays. In contrast, our current report and the report on RhCMV employed synthetic peptides that do not require additional processing for presentation to CD8+ T cells. Since stimulation of CD8+ T cells with live vaccinia virus relies upon the infection, expression and processing of antigen by the cells in the test sample, it is possible that the CD8+ T cell response was intact but antigen presentation by the cells used to present vaccinia antigens in the *in vitro* assay were defective in the aged monkeys. The authors argued that DCs were not affected by the age of the monkeys; however, they only investigated a limited number of parameters and they did not examine antigen processing through the classical MHC class I pathway. Therefore, we cannot discount a possible role for defective antigen presentation in their *in vitro* stimulation. Another possible explanation for the differences may stem from the nature of the immunogens. MVA is a variant of vaccinia virus that replicates poorly in primate cells, whereas RhCMV and WNV replicate effectively in primate cells. Therefore, effective stimulation of CD8+ T cells responses in the elderly may rely upon the nature of the infectious agent. This will be an important point to consider with regard to vaccine design.

It has been proposed that chronic CMV infection may drive immune senescence due to repeated oligoclonal expansions of CMV-specific CD8+ T cells leading to overpopulation of the memory T cell pool [7], [11], [23], [24], [25] and ultimately limiting the ability of the aging individual to combat previously encountered or novel viral infections [25]. However, a recent report has suggested that the size of the CD8+ T cell compartment may increase with age to accommodate expanding memory T cell populations without depleting CD8+ T cells with other specificities [26]. Interestingly, this phenomenon was not reflected within the peripheral blood where the expanding memory populations increased in frequency at the expense of T cells with other specificities. Rather, the expansion of antigen-specific memory CD8+ T cells was accommodated by increased numbers of CD8+ T cells present within the tissues, suggesting that measures of CD8+ T cell frequencies within the peripheral blood may not accurately reflect the true composition of the CD8+ T cell pool. Although it is relatively easy to measure CD8+ T cells present in the tissues in murine studies, addressing this concept in humans is not trivial. Nevertheless, in light of this recent report, the apparent decline in available naïve CD8+ T cells in the peripheral blood of individuals with evident expansion of CMV-specific CD8+ T cells may not truly reflect a corresponding decrease in the availability of naïve T cells in the lymphoid tissues, where primary responses to viruses are initiated.

Similar to previous reports, we have observed a trend towards higher frequencies of CMV- and EBV-reactive CD8+ T cells in the aged cohort. However, this trend did not achieve statistical significance and not all aged individuals displayed an expanded CMV- or EBV-specific CD8+ T cell pool. Similar results have been reported by others [27], [28]. Importantly, in all of these reports, the functionality of the CMV-specific CD8+ T cells did not change with age (the other reports did not investigate EBV-specific CD8+ T cells). It is notable that all of these reports employed functional analyses to define the CMV-specific CD8+ T cells. In contrast, when CMV- and EBV-specific CD8+ T cells were quantified using MHC multimers, it was noted that dysfunctional populations of CMV- and EBV-specific CD8+ T cells accumulate with age based on tetramer staining and IFN-γ production [29], [30]. The implications of these dysfunctional cells are unclear as these aged individuals successfully control both CMV and EBV infections and, based on our results, are able to mount effective CD8+ T cell responses to novel infections. We noted a number of mid-aged and aged individuals with frequencies of CMV-reactive CD8+ T cells that represented more than 9% (9 subjects) of the circulating CD8+ T cell pool, but we did not observe any relationship between expanded CMV-specific CD8+ T cells and impaired generation of WNV-specific CD8+ T cells, indicating that CMV expansions do not limit the ability of the host to respond to a novel infection, consistent with the report of Vezys et al. [26].

Detailed comparison of the functional CD8+ T cell response between the different viruses (WNV, CMV and EBV) revealed interesting differences in functional profiles, corroborating previous reports examining virus-specific CD8+ T cell immunity in humans [31], [32], [33]. Striking similarities in both phenotype and cytotoxic profile were observed between memory WNV- and CMV-specific CD8+ T cells, despite the fact that the former is an acute infection and the latter is a chronic infection. The majority of WNV- and CMV-specific CD8+ T cells displayed a phenotype consistent with terminally-differentiated effectors (CD45RA+ CD28−) whereas EBV-specific CD8+ T cells were mostly less differentiated (CD45RA− CD28+) (data not shown). Consistent with the phenotype and the differentiation status, CMV-specific CD8+ T cells produced high levels of GrB and perforin but failed to produce IL-2, whereas EBV-specific CD8+ T cells failed to produce perforin and had less GrB but significantly more IL-2 (Figure 5A); a similar dichotomy in the production of perforin and IL-2 was described in our previous work with a smaller cohort of patients [33]. This is consistent with previous reports that show the expression of cytotoxic enzymes is related to cellular maturity, such that CD45RA+/− CD28− cells express high levels of cytotoxicity due to highly differentiated phenotype and CD45RA+/− CD28+ T cells express little cytotoxic attributes [33], [34], [35], [36].

Collectively, we demonstrate here that aging individuals are capable of mounting polyfunctional memory CD8+ T cell responses to a novel pathogen, which has significant implications for vaccine development for the elderly. Most of our current understanding on the relationship of aging to vaccination has relied upon measurements of antibodies following vaccination and it is clear that the serological response in the elderly is attenuated [1], [2], [3]. In striking contrast, our results described herein and in our previous report [16] reveal that the aged can mount a robust, polyfunctional CD8+ T cell response to novel pathogens while sustaining a robust polyfunctional responses to chronic infections. Collectively, our data suggest that vaccination in older humans should focus on CD8+ T cell immunity and that live vaccines should be considered as the platform of choice. The results presented here entice our curiosity and desire to better understand the aging immune system for the purpose of developing much needed vaccines for our greatly expanding aging population.

## Materials and Methods

### Ethics statement

This research was approved by the Hamilton Health Sciences/McMaster Health Sciences Research Ethics Board that operates in compliance with the ICH Good Clinical Practice Guidelines and the Tri-Council Policy Statement: Ethical Conduct for Research Involving Humans and Division 5 Health Canada Food and Drug Regulations. All patients in this study provided informed written consent.

### WNV patient cohort and PBMC preparation

Seventy-two patients were enrolled into the study following detection of serum WNV IgM by public health laboratories after presentation of WNV-related symptoms. Serology for WNV was assessed by plaque reduction neutralization assay as described previously [37]. Recruitment of patients occurred over a period of 5 years (2003–2007). This trial was reviewed and approved by the Research Ethics Board at McMaster University.

Patients were entered into our study within 1 month following symptom onset (median = 30 days, ranging from 4–100 post symptom onset) and blood was collected on the first visit (baseline sample) and once every month thereafter for a period of one year. Twenty-five patients were contacted 2–4 years post symptom onset and their blood was collected at convalescence of disease. The population consisted of 37 men and 35 women ranging in age from 19 to 85 years. Patients were subdivided into three cohorts for these experiments based on age; young <40 years of age, mid-aged 41–59 years, and aged >60 years of age (Table S1).

Blood samples were drawn into heparanized tubes and PBMC were isolated from the blood by centrifugation on Ficoll (Amersham Pharmacia). PBMC were cryopresrved in RPMI 1640 containing 12.5% human serum albumin (Sigma-Aldrich) and 10% DMSO according to the method described by Disis et al. [38].

### Peptide stimulations

WNV peptides used for the stimulation of PBMCs were identified previously [16] and 13 of commonly immunogenic peptides were pooled together for the purpose of having a single WNV stimulation that would encompass the vast majority of reactivities within the cohort. Peptides were either deconvoluted to a minimal epitope of 8–9 amino acids or were uses as a 15-mer. CMV stimulation consisted of 168 identified CMV-specific CD8+ T cells epitopes pooled into a single pool. Likewise, EBV stimulation consisted of 91 identified EBV-specific CD8+ T cell epitopes pooled together into a single pool.

### Intracellular cytokine staining

PBMC were thawed and placed immediately into 37°C pre-warmed complete RPMI (Invitrogen) supplemented with 10% fetal bovine serum (FBS), 2 mM L-glutamine, 50 µM 2-ME, 10 µM HEPES, 100 U/ml penicillin, and 100 µg/ml streptomycin. Thawed PBMC were cultured overnight at 37°C incubator. The cells were subsequently harvested, counted, and viability was assessed by trypan blue exclusion. Cells were aliquoted (2–2.5×10^6^ cell/well) into round-bottom, 96-deep-well plate (Costar); peptides were added to a final concentration of 2 µg/ml and were incubated for 1 hr at 37°C. DMSO diluted in cRPMI was used as a peptide-non-specific negative control. Brefeldin A and Monensin A (BD Biosciences) were added to the cell/peptide mixture as per manufacturer's instructions and were incubated for an additional 4 hrs. At this point, cells were pelleted and washed in 10 µM EDTA. The cells were first stained with Near IR viability stain (Invitrogen, Molecular Probes) and subsequently with different antibody cocktails depending on the analysis. Cytokine analysis cocktail comprised of anti-human CD3-Qdot 605 (Molecular Probes), CD8-Alexa flour 700, CD45RA-PE Texas red (Beckman Coulter), CD28-PE Cy5, CD4-Pacific Blue, CD14-Pacific Blue, CD19-Pacific Blue surface antibodies. Cells were then permeabilized with Cytofix/Cytoperm (BD Biosciences), and intracellular cytokines were identified using anti-human IFNγ-APC, TNFα-FITC, and IL-2-PE [Note: all flow cytometry reagents were obtained from BD Biosciences unless otherwise specified]. Fluorescence data were acquired using LSRII flow cytometer (BD Biosciences) and 500,000–1 000,000 events based on the live lymphocyte gate were collected per sample. Data were analyzed using FlowJo. A positive response was measured as the IFN-gamma frequency greater than 0.05 and three fold above DMSO background.

### Cytotoxic analysis

PBMCs were cultured and stimulated as described above however in addition to the stimulatory peptides, anti-human CD107a-PE conjugated antibody (BD Biosciences) was added to the cells at the beginning of the stimulation for 1 hr. Brefeldin A and Monensin A (BD Biosciences) were added to the cell/peptide/CD107a mixture as per manufacturer's instructions and were incubated for an additional 4 hrs. The cells were subsequently stained with a Near IR viability stain (Invitrogen, Molecular Probes) as per manufactures instructions followed by the cytotoxic antibody cocktail: anti-human surface antibodies [CD8-PerCP Cy5.5 (eBiosciences), CD4-Alexa Flour700 (BD Biosciences), CD19 and CD14-Alexa Flour 700 (eBiosciences)] and intracellular anti-human antibodies [IFNγ-APC, Granzyme B-FITC and Perforin-Pacific Blue (conjugated to Pacific Blue in house using standard conjugation protocols)]. The perforin antibody detects *de novo* as well as pre-formed perforin and when used in conjuction with IFNγ following in-vitro peptide simulation we are able to determine the frequency of *de novo* formed perforin only. Fluorescent data was acquired using the LSR II as described above.

### Phenotyping of PBMCs

An aliquot of thawed patient PBMCs (0.5–1×10^6^ cell/stain) was used for the purpose of phenotyping the cells. Cells were stained in round-bottom 96-well plates with anti-CD3-APC-H7, CD8-Alexa Flour 700, CD4-Pacific Blue, CD45RA-PE Texas Red (Beckman Coulter), CD28-PE, CD57-FITC, and anti-CCR7-PE Cy7 [Unless otherwise stated all antibodies were purchased from BD Biosciences]. Analysis of surface marker staining was done by LSR II flow cytometer and data was analyzed using FlowJo software.

### FLOCK analysis

FLOCK is an automated computational approach publically available at the Immunology Database and Analysis Portal – ImmPort (www.immport.org), which utilizes algorithms and density-based clustering to identify cell subsets. FLOCK analysis is comprised of five components: data preprocessing, grid-based density clustering, cross-samples comparison, result visualization, and population statistics calculations. Detailed methodology for FLOCK analysis can be found in [18]. In summary, binary .fcs files specifically gated on live/singlet/CD3+/CD8+ CD4−/IFNγ+ events were converted to tab-delimited ACSII text format and exported from FlowJo (Tree Star) in a data matrix file. Samples (6–7 months post WNV symptom onset) were considered positive if following peptide stimulation they expressed IFNγ frequency above 0.05 and 3 fold above DMSO background and consequently were included in the FLOCK analysis. This was the means by which our data was normalized. The exported CD8+ IFNγ+ events were than subjected to density-based grouping based on expression of IFNγ, TNFα and IL2 for determination of cytokine populations; and IFN-γ, CD107a, GrB and Perforin for determination of cytotoxic populations depending on the distances between each point and where its coordinates lie in the defined grid. FLOCK identified 16 cell populations: 6 defining cytokine populations and 10 defining cytotoxic populations (Figure 3). Population centroids (the average of coordinates of a given set of points) were applied to multiple samples in a cross-sample analysis to enable population comparisons between WNV, CMV, and EBV-specific CD8+ memory T cell populations.

### Statistical analysis

The data are presented as mean values. Simple descriptive statistics (means, standard deviations, Students t test and regression analysis) were calculated using GraphPad Prism version 1.0. Box and whiskers plots are calculated at 95% confidenced interval and generated using GraphPad Prism version 1.0.

The large data set comprising functional population frequencies was analyzed by Principal Component Analysis (PCA), which is a linear technique that transforms data of interrelated variation of the data set in reduced dimensionality [19]. To graphically reveal clustering, multi-collinearity and outliers of our data set following PCA we used a biplot consisting of top −2 PCs. The biplots show both the samples and features of the data set, where each sample is displayed as a point in a two-dimensional plane, and each functional population (defined by FLOCk clustering) is presented as a vector. The length of each vector indicates the approximate variance of the specific functional population. The distance between two points is an approximate of the Euclidean distance between their associated functional phenotype. Thus, samples that cluster together are interpreted as similar. Conversely, observing no clustering of points implies very little similarity among the data points. The correlation between any two functional phenotypes can be approximated by the angles between them, where angles of 90 or 270 degrees apart show correlations approaching zero, and angles of 0 or 180 degrees show a correlation of 1 or −1, respectively.

The Kolmogorov-Smirnov (KS) test was used to determine whether WNV, CMV or EBV-specific CD8+ T cell functional phenotypes were drawn from the same or different distributions. The KS statistic quantifies a distance between the empirical distribution functions of two samples and is based on the null hypothesis that the samples are drawn from the same distribution if the p value approaches 1.

## Supporting Information

Table S1 **Patient characteristics and reactivities.** All patients associated with the studies in this manuscript are listed in this table using their anonymous identifiers. Age and sex (M = male, F = female) are listed. The percentage of CD3+CD8+ T cells that produced IFN-γ in response to the WNV, EBV, and CMV peptide pools is shown. Specimens used for measuring the virus-specific responses listed in this table were obtained 6–7 months following the onset of WNV infection. A positive response was defined as greater than 0.05% of CD3+CD8+ T cells and 3 fold above DMSO background. All the data shown in this table have been corrected for the DMSO background. Responses that were defined as negative are indicated by an asterisk (*).
(DOC)
