# Supplementary material for: The Polyfunctionality of Human Memory CD8+ T Cells Elicited by Acute and Chronic Virus Infections Is Not Influenced by Age
Source: PLoS Pathog. 2012 Dec 13;8(12):e1003076. doi: 10.1371/journal.ppat.1003076 (PMC3521721; doi:10.1371/journal.ppat.1003076)
Supplement: Table S1 — Patient characteristics and reactivities. All patients associated with the studies in this manuscript are listed in this table using their anonymous identifiers. Age and sex (M = male, F = female) are listed. The percentage of CD3+CD8+ T cells that produced IFN-γ in response to the WNV, EBV, and CMV peptide pools is shown. Specimens used for measuring the virus-specific responses listed in this table were obtained 6–7 months following the onset of WNV infection. A positive response was defined as greater than 0.05% of CD3+CD8+ T cells and 3 fold above DMSO background. All the data shown in this table have been corrected for the DMSO background. Responses that were defined as negative are indicated by an asterisk (*). (DOC) [file ppat.1003076.s001.doc]

**S1.** **Patient Characteristics and Reactivities.** All patients associated with the studies in this manuscript are listed in this table using their anonymous identifiers. Age and sex (M = male, F = female) are listed. The percentage of CD3+CD8+ T cells that produced IFN-γ in response to the WNV, EBV, and CMV peptide pools is shown. Specimens used for measuring the virus-specific responses listed in this table were obtained 6-7 months following the onset of WNV infection. A positive response was defined as greater than 0.05% of CD3+CD8+ T cells and 3 fold above DMSO background. All the data shown in this table have been corrected for the DMSO background. Responses that were defined as negative are indicated by an asterisk (*).

|  |  |  | **% IFN-γ+ of total CD3+ CD8+ cells** | | |
| --- | --- | --- | --- | --- | --- |
| **Patient I.D.** | **Age** | **Sex** | **WNV** | **CMV** | **EBV** |
| 77408 | 19 | F | 0.811 | 5.220 | 1.121 |
| 77407 | 23 | F | 0.154 | 0.007* | 0.254 |
| 44402 | 24 | M | 0.365 | 0.006* | 0.009* |
| 77302 | 29 | M | 0.779 | 0.001* | 0* |
| 77304 | 30 | F | 0.486 | 0.004* | 0.456 |
| 77323 | 30 | F | 0.105 | 0.021* | 0.335 |
| 77421 | 30 | F | 0.124 | 0.244 | 0.041* |
| 10202 | 32 | M | 1.270 | 0.011* | 0.130 |
| 55308 | 33 | F | 0.066 | 0.012* | 0.804 |
| 77423 | 35 | M | 0.395 | 0.135 | 0.245 |
| 55313 | 36 | F | 0.135 | 0.017* | 0.250 |
| 77404 | 36 | F | 0.333 | 0.046* | 0.353 |
| 77413 | 37 | M | 1.542 | 0.07* | 0.382 |
| 60002 | 38 | M | 0.250 | 1.650 | 0.520 |
| 5201 | 39 | M | 0.273 | 0.007* | 0.03* |
| 77324 | 39 | M | 0.174 | 0.008* | 6.040 |
| 77419 | 39 | M | 1.449 | 0.015* | 0.289 |
| 55420 | 40 | F | 0.120 | 1.260 | 0.270 |
| 77309 | 40 | F | 0.153 | 1.000 | 0.783 |
| 77310 | 40 | M | 0.185 | 2.285 | 0.565 |
| 77321 | 40 | F | 0.042* | 0.053* | 0.691 |
| 77315 | 41 | F | 0.238 | 0* | 0.308 |
| 77319 | 41 | F | 0.150 | 0.014* | 1.120 |
| 10201 | 45 | F | 0.250 | 4.201 | 3.120 |
| 77312 | 45 | F | 0.188 | 0.018* | 2.198 |
| 77316 | 45 | M | 1.178 | 9.118 | 3.088 |
| 77401 | 45 | F | 0.129 | 3.939 | 0.499 |
| 77505 | 46 | M | 0.255 | 0.014* | 0.605 |
| 77301 | 47 | M | 0.350 | 0* | 0.06* |
| 77327 | 47 | M | 0.100 | 13.190 | 0.720 |
| 77411 | 47 | F | 0.327 | 0* | 0.004* |
| 55405 | 47 | F | 1.115 | 0.005* | 0.042* |
| 77320 | 49 | F | 0.083 | 0.004* | 0.502 |
| 55410 | 51 | M | 4.254 | 3.404 | 10.784 |
| 55413 | 51 | M | 0.504 | 0.009* | 17.184 |
| 44410 | 52 | M | 0.964 | 0.027* | 0.724 |
| 77422 | 52 | M | 1.285 | 0.04* | 1.155 |
| 77307 | 55 | F | 1.704 | 19.674 | 0.022* |
| 77313 | 55 | F | 0.530 | 0.02* | 2.720 |
| 77317 | 55 | M | 0.438 | 13.398 | 1.638 |
| 77322 | 55 | F | 0.046* | 0.088 | 0.228 |
| 55414 | 57 | M | 0.154 | 0.194 | 0.444 |
| 77303 | 57 | F | 1.740 | 1.087 | 1.507 |
| 77326 | 57 | F | 0.575 | 0* | 0.335 |
| 77331 | 57 | M | 0.329 | 0.959 | 1.829 |
| 77424 | 58 | F | 0.426 | 3.226 | 0.196 |
| 55408 | 60 | F | 0.211 | 0.008* | 0.481 |
| 77332 | 60 | F | 0.026* | 1.093 | 0.643 |
| 77502 | 60 | F | 0.263 | 0.004* | 0.753 |
| 77403 | 61 | F | 0.155 | 9.136 | 0.027* |
| 77412 | 62 | F | 0.00* | 9.560 | 4.860 |
| 55407 | 63 | M | 0.197 | 0.03* | 1.897 |
| 77402 | 63 | F | 0.579 | 0.549 | 0.229 |
| 11201 | 64 | M | 0.690 | 6.490 | 8.280 |
| 55310 | 64 | F | 0.257 | 0.015* | 5.057 |
| 77311 | 64 | M | 0.350 | 0.031* | 0.290 |
| 77405 | 64 | M | 2.339 | 0* | 2.189 |
| 44405 | 65 | F | 0.108 | 9.708 | 0.158 |
| 60003 | 65 | M | 0.200 | 0.100 | 0.053 |
| 7001 | 66 | M | 0.886 | 0.015* | 2.516 |
| 77425 | 66 | M | 0.663 | 0.001* | 0.007* |
| 51002 | 67 | M | 0.374 | 0.154 | 0.079 |
| 77420 | 67 | M | 1.238 | 8.738 | 0.258 |
| 55311 | 69 | M | 0.003* | 2.660 | 0.340 |
| 60001 | 71 | M | 0.271 | 7.241 | 0.461 |
| 51001 | 73 | M | 0.199 | 3.249 | 2.039 |
| 55401 | 75 | M | 0.147 | 15.157 | 3.787 |
| 77329 | 77 | M | 0.291 | 3.101 | 0.371 |
| 55316 | 78 | F | 0.166 | 34.786 | 2.786 |
| 77406 | 80 | M | 1.203 | 0.032* | 2.243 |
| 77409 | 81 | F | 0.274 | 12.824 | 1.934 |
| 9001 | 82 | M | 0.050 | 7.887 | 0.607 |
